# Patent Landscape of Automated Systems for Personalized Health Management (ASHM): Features, Shortcomings, and Implications for Developing an Optimal ASHM

**DOI:** 10.3389/fdgth.2021.579936

**Published:** 2021-02-11

**Authors:** Andrey Martyushev-Poklad, Dmitry Yankevich

**Affiliations:** Federal Research and Clinical Center of Intensive Care Medicine and Rehabilitology, Moscow, Russia

**Keywords:** health management system, preventive medicine, personalized medicine, continuity of patient care, patient-centered care (MeSH term), lifestyle interventions, behavior modification, decision support systems

## Abstract

The current struggle of national health care systems against global epidemic of non-communicable diseases (NCD) is both clinically ineffective and cost ineffective. On the other hand, rapid development of systems biology, P4 medicine and new digital and communication technologies are good prerequisites for creating an affordable and scalable automated system for personalized health management (ASHM). The current practice of ASHM is better represented in patent literature (36 relevant documents found in Google Patents and USPTO) than in scientific papers (17 documents found in PubMed and Google Scholar). However, only a small fraction of publications disclose a complete self-sufficient system. Problems that authors of ASHM aim to address, methodological approaches, and the most important technical solutions are reviewed and discussed along with shortcomings and limitations. Technical solutions for ASHM currently commercialized or described in literature generally fail to enable practicable, scalable and affordable automated and individualized screening, monitoring, prevention and correction of human health conditions. They also fail to provide a decision support system to patients that would help effectively prevent major NCD and their complications, be accessible and cost effective, consider individual lifestyle factors and involve patients in management of their individual health. Based on analysis of the literature, models of health and care, we propose conceptual framework for developing an ASHM that would be free from the mentioned problems.

## Introduction

In the context of global epidemic of non-communicable diseases (NCD) and active progress of digital technologies, there is a growing understanding that automated health management systems could be of great help to improve patient outcomes and reduce health care costs.

Health management is essentially the meaning and the major content of medical practice. However, if we compare the definition of health given by the World Health Organization (1) with goals and objectives of real clinical practice, it is obvious that today's health care focuses on a narrow range of health continuum (2, 3)—namely, on managing diseases. This is true not only for healthcare institutions but also for companies involved in digital health: only 23.8% of them focus on prevention, while the rest develop products and services for diagnostics and treatment of health conditions (4).

With all the above, there is a considerable gap between general theoretical understanding of the causes behind NCDs and the instruments and interventions that health professionals practice on daily basis. Thus, for example, it is generally understood that more than 80% of health status is determined by lifestyle factors (5); however, worldwide the major instrument of health interventions are not lifestyle interventions but pharmacotherapy.

From economic viewpoint, preventive measures are much more cost effective (in terms of health improvement per invested resources) than the treatment of disease complications. This is why “reactive” strategy of treating already existing NCDs and their complications will always face scarcity of resources. Another major factor of scarcity is allocation of roles in today's prevalent model of health care: the burden of decision making, interventions and responsibilities is born by the doctor, whereas the most efficient measures leading to health improvement are closely related with the patient's behavior, with his/her ability to manage one's own health.

The trend toward a more efficient model of health care is connected with the paradigm of “P4 medicine”—predictive, preventive, personalized, and participatory (2, 6). An important prerequisite of improving healthcare efficiency are digital and communication technologies (e-Health, including telemedicine) and patient-centered technologies (individual wearable devices, health trackers, etc.) used to address patients' needs across the whole continuum of health care (7–9).

However, at the level of scalable technologies, the new more effective paradigm of P4 medicine is far from implementation.

This paper reviews current industry practices and technical solutions in automated management of individual health proposed in marketed services and patent literature: (1) the problems that they address, (2) their actual capabilities, and (3) their limitations. We also discuss promising directions and potential framework for developing an optimum automated system for personalized health management (ASHM).

## Sources of Information About Current Practices in Automated Systems for Personalized Health Management and Search Criteria

There is a wide range of technical solutions on the market for managing patient's medical data. These include medical information systems, health information systems, hospital information systems, clinical information systems. They are all intended to manage electronic medical records or electronic health records and support clinical decision making. At the national level, such systems serve to evaluate and improve the quality of health care and to manage the whole health care system (10, 11). However, they cannot solve the tasks of managing the health of individual patients, the more so in automatic mode.

Before going into analysis of available data on ASHM we need to first define this term. The field that it belongs to is Digital Medicine, Digital Health and Care. “Digital medicine” describes a field concerned with the use of technologies as tools for measurement, and intervention in the service of human health (12). According to Ejehiohen (13), “digital health” is an improvement in the way healthcare provision is conceived and delivered by healthcare providers through the use of information and communication technologies to monitor and improve the well-being and health of patients and to empower patients in the management of their health and that of their families. Another very broad definition was given by Frost and Sullivan (14): “Digital health refers to a vast market of information technology applications, platforms and services leveraged by healthcare providers, payers, med tech and life sciences companies, patients, and consumers.”

We see ASHM as an integrated, self-sufficient technology solution designed to solve a wide range of tasks of long-term personalized health preservation, disease prevention and treatment through automated collection, storage, management and exchange of health-related information.

ASHM can be considered as one of possible information technology applications within digital medicine and digital health fields. This should be an integrated solution (product or service), so ASHM has to involve to certain extent all four aspects of digital health according to Deloitte (15): Telehealthcare (with remote monitoring and consultation), mHealth (smart mobile devices and applications), data analytics (for data-driven decision making), and digitized health systems (management, storage, and exchange of electronic health records).

We are developing such an integrated solution, so this review was initially plotted and designed as an analysis of prior art for a patent application on ASHM. Therefore, its main purpose is to reveal and systematize the key features of published potentially scalable relevant technical solutions having a commercial potential. That is, we focus not on just any technology of digitalization or automation of health care, health measurement or health intervention. We are rather interested in a self-sufficient automated system of collecting, processing and use of health-related information that should provide a measurable clinical result to an unmet problem or need of a real customer (patient). Our idea was to understand both the organizational model and technical IT solutions that are able to give a new result in the form of restoring or improving measurable parameters of health, along with understanding the shortcomings and gaps of the existing solutions.

Therefore, we had quite strict and narrow requirements to the documents we were looking for; they had to describe:

(1) an integrated (self-sufficient) system capable to solve major tasks of health care continuum, not only improve certain steps of an existing system;
(2) the scientific and organizational model of managing health that the system is based on (namely, biomedical or biopsychosocial model);
(3) the types of information collected, the purpose and mode of collection;
(4) the way this information is processed or analyzed;
(5) the logic of decision making regarding patient's health, and the type of health-related decisions it supports;
(6) measurable health-related results it provides as the consequence of better decision making.

Based on the above criteria, we used primarily the following key words that had to be present in the summary of searched documents: “system,” “health,” “automated,” “management,” “human,” “personal*” (for example, “automated” AND “health management system”). All documents matching these key words were further analyzed for the criteria described above.

In our search of the current practices, we first performed search in two open databases: PubMed and Google Scholar. With PubMed database (https://pubmed.ncbi.nlm.nih.gov/) we used key words “health management system,” with and without “automated.”

With probe and error we chose the most productive keywords for search in Google Scholar (https://scholar.google.com/): “human” AND “personal*” AND “health management system.” We opened all sources containing the whole phrase “health management system” and checked them for the above criteria of a self-sufficient ASHM.

We understand that with technologies bearing a commercial potential it is a routine strategy that their authors use a specific logic of bringing them to public. To avoid anticipation of novelty of their products and technologies, inventors tend not to publish them in peer reviewed journals but instead disclose them in the first place through analysis of prior art and detailed description of the invention in patent application. Also patent sources were somewhat preferred to non-patent literature due to other reasons:

1) Patent applications usually don't have space and format limitations inherent in peer reviewed research papers, as well as quality of data supporting the results the system provides (i.e., the system can be described as early as at the stage of idea);
2) Patent applications have to provide adequate technical description of the claimed invention to meet patentability criteria;
3) Patent applications focus on practical results and advantages that the invention can provide, and outline their distinctive features from similar solutions; on the other hand, they can disclose ideas not yet put into practice.

We analyzed granted patents and published patent applications covering systems for health management. Keyword search in Google Patents (https://patents.google.com/) was performed with keywords in Abstract (AB = “health management system” AND “human” AND “personal*”). We then analyzed separately patent documents filed with major national patent offices (USPTO, EPO, in Japan, China, and Korea). To make sure that all potential patent documents are analyzed, we also performed search in the database of US Patent and Trademark Office (USPTO, http://patft.uspto.gov/) over the whole available time frame (2001-present for applications, 1976-present for full text patents) with the following broad key words in claims: “health management.”

In rating the relevance and quality of the found published descriptions on ASHM, both in patent and non-patent literature, we proceeded from understanding that the currently prevalent biomedical (BM) disease-centered model of health and care is effective in managing acute disease and urgent conditions. On the other hand, chronic conditions cannot be effectively managed without use of the biopsychosocial (BPS) patient/person-centered model of health, disease and care, also known as “P4 medicine” (2, 6).

Meeting criteria of a self-contained HMS meant that in addition to just claiming a HMS the document had to describe the scientific model behind health management, the target patient audience, the types of collected data and/or principles of data collection, the principles of data processing and analysis, the logic of decision making and health intervention, and/or measurable health-related results. Automation of at least one step of the whole process was mandatory.

## Health Management Systems Described in Non-patent Literature

PubMed retrieved only 5 results with search string “health management system” AND “automated.” This either means that the authors of peer reviewed papers use different key words to describe their subject in the Abstract, or indeed only very few papers focus on the broad problem of designing a robust automated system for managing individual health. The search “health management” and “system” and “automated” gave 54 results, only 3 of them being relevant to our narrow criteria. The search “health management system” retrieved 151 results, 19 of them relevant to our understanding of a self-contained ASHM. Analysis of their relevance to the criteria set in section Sources of Information About Current Practices in Automated Systems for Personalized Health Management and Search Criteria is summarized in Table 1.

**Table 1:** Publications on automated systems for personalized health management found in patent and non-patent literature ranked by their correspondence to criteria for a self-sufficient integrated solution (criteria are described in section Sources of Information About Current Practices in Automated Systems for Personalized Health Management and Search criteria).

**References**	**Criteria set for a ASHM that description has to meet**					
	**Integrated**	**BM/BPS model**	**Info flow**	**Info analysis**	**Provide decision making**	**Provide results**
(16–19)	+	BPS	+	+	+	–
(20)	+	BPS	+	–	+	+
(21)	+	BPS	–	–	+	–
(22–28)	+	BPS	+	–	–	–
(29, 30)	+	BM, BPS	+	–	–	–
(31)	–	BPS	+	–	+	+
(32)	–	BPS	+	+	–	+
(33)	–	BPS	+	+	–	–
(34–36)	–	BPS	+	–	–	–
(37)	–	BPS	–	–	+	–
(38)	+	BM	+	+	+	+
(39, 40)	+	BM	+	–	–	–
(41–45)	–	BM	+	+	+	+
(46–48)	–	BM	+	+	+	–
(49–61)	–	BM	+	+	–	–
(62)	–	BM, BPS	+	–	–	–
(63–67)	–	BM	+	–	–	–
(68)	+	BM	–	–	–	–

*BPS, biopsychosocial (patient/person-centered) model; BM, biomedical (disease-centered) model of health, disease and care*.

Our Google Scholar search with keywords “human,” “personalized,” and “health management system” retrieved 694 sources without patents, and 2,860 sources with patents—i.e., patents accounted for 76% of search results. Despite the term “human,” many found papers covered health management of non-human systems (technical or animal). In addition to peer reviewed papers, the sample also contained conference proceedings and books. We opened and read all found entries with the phrase “health management system.” Out of 694 Google Scholar entries some overlapped with PubMed results, and only 3 represented additional publications relevant to a self-contained ASHM, they are included in Table 1. All sources mentioned in Table 1 are further briefly commented below.

The earliest article, by Risk et al. (49), describes Anscore Health Management System for evaluation and interpretation of heart rate variability, developed by Boston Medical Technologies (USA). Although presented as a “health management system,” it can only be used for early detection and monitoring of autonomous dysfunction in different neurological disorders. On the other hand, heart rate variability measured by a wearable device can be a useful instrument in self-monitoring and self-management of a chronic condition (69), hence this is a promising universal component for an ASHM.

Lim et al. (41) describe a concept of ubiquitous healthcare (“u-healthcare”) as an individualized health management system for diabetic patients. The paper demonstrates how advanced technologies enable individualized approach to control blood glucose, ease health education and patient involvement, as well as improve patient autonomy in health management. Outpatients can independently monitor blood glucose levels, and glucometer transfers the readings through mobile phone into automated clinical decision support system (CDSS). Based on the readings, the CDSS reminds patients about regular measurement of glucose levels and give instructions on medications through text messages. This approach improves long-term (6 month) outcomes in diabetes assessed by HbA1c levels.

A more general description of u-healthcare system (42–45) reveals its 3 basic components: (1) a portable device for reading patient's biological parameters (blood glucose, ECG, EMG, blood pressure, oxygen saturation, and body temperature) and/or diet diary, (2) a smartphone application and a special device to transfer the readings to a server, and (3) a server monitoring and analyzing the readings. The described u-healthcare system has at least the following limitations: (1) It can only be used in patients with established NCD diagnosis. (2) It monitors mostly biological parameters. (3) It practically fails to involve correction of lifestyle factors, the major cause of NCD progression. Generally the idea of u-healthcare is a great step forward in adapting healthcare to patients' needs and introducing self-health management. However, as proposed so far it has very limited utility.

An article by Lau et al. (29) and three other papers by this team from New Zealand describes a new eHealth solution, Web-based personally controlled health management system (PCHMS, *Healthy.me*), and its utility in involving mostly young adults (university students and staff) in more effective help-seeking behaviors for emotional well-being, raising awareness of personal well-being, and mastering certain self-care tools. Novel features of this solution included personal health records, social networking (forum) and a diary encouraging self-reflection and self-awareness. The major advantages of PCHMS reported by Lau et al. are: it is closer to the biopsychosocial model of well-being; it extends continuum of health and continuum of care that it can cover; it raises awareness and involvement in help-seeking and self-care activities; this is a personalized approach; it focuses on disease prevention and early interventions. However, the proposed system lacks some features crucial for its long-term workability: (1) It is so far used for narrow disease-centered purposes (it is currently recruiting patients with asthma diagnosis) and with time limits (a 12-month study); (2) It doesn't provide a comprehensive decision making support tool and relies much on a medical professional.

Paper by Dhillon et al. (22) covers in detail a concept of developing a patient-centered HMS with a focus on psychosocial aspects of well-being in senior patients. However, it lacks essential features of information analysis and decision making, hence it cannot provide measurable health-related results to its users.

Gutenbrunner et al. (38) describe a HMS to improve workability of medical staff with musculoskeletal conditions. Its limitations are no use of digital medicine and a narrow disease-centered focus.

Bloss et al. (50) describe an online HMS with active use of wearable devices to monitor parameters of an established chronic health condition; the study focuses on health care costs and provides no measurable health-related results.

Soh et al. (63) describe a mobile care system for advanced cancer patients; its functionality is limited to self-monitoring.

Hsieh et al. (64) describe a patient-centered mHealth application for self-assessment and medication management; its utility is limited to better use of pharmacological treatments.

Neubert et al. (65) describe a system for integration of data from multiple sensors and data sources that can be used in preventive and occupational medicine. But this system lacks an integrated approach to health management, so it is a purely technical solution.

Nedungadi et al. (16) describe a patient-centered health awareness and monitoring system for rural areas; it is based on physiological data obtained with wearable devices, processed through an e-health platform to support individualized doctor's decision making on emergency interventions, preventive care, and lifestyle interventions (including Ayurveda and yoga).

Gandarillas and Goswami (23) propose methodology of building a home-based and community-centered Integrated Healthcare Management System with special focus on biopsychosocial model and aging patients with chronic disease. It describes in detail the possible organizational structure of services and their communication structure. With an in-depth overall design, it lacks specific algorithms of data collection, analysis and use in decision making.

A very important aspect of health management, namely health coaching, is covered by Andreou and Raspopoulos (24). With detailed concept description, this paper however lacks specific technologies and algorithms needed to produce specific results.

## Health Management Systems Described in Google Patents and the USPTO

Search in Google Patents (as of 2021-01-15) provided a total of 302 results matching key words “health management system,” “human,” and “personal*” in Abstract, 12 of them relevant to an integrated HMS: 31 in WIPO (3 relevant), 24 in USPTO (4 relevant), 6 in EPO (0 relevant), 71 in JPO (1 relevant), 148 in Chinese Patent Office (3 relevant), 22 in Korean Patent Office (1 relevant).

A separate patent search in the USPTO database with key words in claims “health management” retrieved 490 applications (as of 2020-06-03); “health management” and “human” retrieved 36 applications. Out of all found records (partially overlapping with Google Patent search), we selected 204 that were actually relevant to human health. The rest were either focused on non-human systems or business operations. They also had to meet criteria of a self-sufficient system, that's why many local methods and devices used for managing health and data management systems were excluded. A total of 29 documents appeared relevant to an integrated HMS. All these documents are ranked for criteria of ASHM in Table 1 and each one is analyzed or briefly commented below.

In this section, we outline authors' motivation to develop an AHMS (problems they address), and briefly describe technical solutions within two models of care, disease-centered reactive and patient-centered preventive models.

### Problems That Health Management Systems Are Designed to Address and Proposed Solutions

The problems, factors and objectives that authors of health management systems address are summarized in Table 2 along with the essence of proposed solutions.

**Table 2:** Problems that a system of personal health management should address and certain common technical solutions for them found in automated health management systems (AHMS) proposed in patent literature.

	**Groups of problems**	**Proposed solutions**
1.	Reactivity of health care: Lack of planned, regular, continuous proactive interaction with the patient Lack of adequate feedback from the patient, including feedback concerning assessment of health state and its changes over the course of treatment or follow-up Too late interventions due to lack of opportunity for mass screening, monitoring, and prevention	Questionnaires to evaluate current health condition and NCD risks (17, 18, 31, 32, 35, 39, 47) Individualized questionnaires (37) Questionnaires to evaluate treatment efficacy (35) Consideration and analysis of early premorbid changes of health (17) Remote collection, transfer and analysis of information about symptoms and lifestyle factors (17, 18, 31, 32, 34, 36, 39, 57, 66, 68) Automated collection, exchange and analysis of patient's objective data (body measurements, laboratory tests) (17, 33, 35, 36, 39, 46–48, 52, 53, 56, 68) Use of portable or wearable device to evaluate physiological parameters, including laboratory data (17, 35, 39, 46, 48, 53, 56, 68, 70)
2.	Insufficient productivity of the doctor: limited number of patients that he/she can follow simultaneously	Automated use of official clinical guidelines or standard programs to propose unified measures of risk reduction (32, 35, 46–48) Automated use of scientific publications to develop individualized recommendations (31, 33, 35, 37) Opening access for different doctors to patient's profile with information of health state and treatment schedule (67) Telemedicine-based interaction between patient and doctor (31, 39) The use of a digital model of physiology and machine learning-based artificial intelligence (68)
3.	Lack of infrastructure for patient education, information and motivation to behavior change	Modification of patient's behavior through information and lifestyle recommendations (17, 18, 31, 33, 34, 37, 39, 46–48, 68) Interactive representation of educational information to the patient depending on the feedback (51) The use of community to motivate patients to lifestyle changes (through gamification) (62) Graphical display of signs and symptoms; matching them with patient's behavior (36, 58) Estimation of expected life span and healthcare expenses as a motivation tool (52)
4.	High overall cost of health care, partially due to lack of prevention	Management of disease treatment (for diabetes) in home setting with complete cycle of data processing: collection, analysis, recommendations on patient's activities, control of compliance, evaluation of results (60) Keeping an analog of electronic medical record with individual planning and control of examinations and interventions (in a particular disease, diabetes) (61)
5.	Insufficient consideration of patient's life context, beliefs, sources of resistance and motivation, evaluation of his/her condition Overall insufficient individualization of lifestyle guidelines and recommendations	Individualized questionnaires (37) Evaluation of genetic polymorphisms for measuring NCD risks (32, 33) Consideration of patient's emotional state (stress, depression) (57) The use of integrative medicine (17, 21) Integration with third party data about patient's locations and behavior in social networks (18)
6.	Insufficient patient's autonomy in self-diagnosing and prevention	Teaching physical exercise with the use of telemonitoring and reference video (54, 55) The use of coaching (17, 31, 32, 39) Providing video instruction about necessary actions (based on individual goals) (51) Daily plan with reminding the patient about necessary measures (18, 56, 59)
7.	Gaps is continuum of care	Long-term follow-up in home setting (17, 18, 31, 39, 51)

A major objective of inventors is to create an automated interactive system that would enable regular remote interaction with multiple patients suffering from NCD in order to educate, inform them on their health state, to motivate behavioral changes related to their health (to modify risk factors of NCD), to improve compliance with medical measures, to monitor health indicators, and to enable early interventions designed to improve health (46, 47).

Management of NCD requires continuous interaction with patients, where costs of follow-up can be reduced by organizing interaction in outpatient setting or at home. Success of such programs depends on effective monitoring of patients' state, detection of abnormalities and early interventions to prevent possible complications which are much more difficult and expensive to treat. The overall success is determined by patients' motivation and their active efforts to modify their lifestyle (51).

The current system of health care is “reactive”; the doctor reacts to patient's phone call or visit to clinic. As the result, the doctor interacts mainly with the most motivated patients or with those in emergency. From the viewpoint of long-term outcomes and health improvement, the most efficient interaction would be “proactive” scheduled contacts with least motivated patients who are not inclined to visit or call the doctor. The least motivated patients often develop emergencies that could have been prevented through planned, “proactive” communication. Therefore, the cost of treating such NCD patients could be reduced by planned interaction and by increasing their motivation to change behavior and lifestyle. Patient's motivation is an important factor that should be considered in the plan of health management (51).

Patients with higher risk of disease complications, those who need additional examination and care, can be timely identified through planned communication; this is necessary to improve the efficiency of care providers (51).

Most of the existing medical information systems are designed to display individual medical data and don't allow the doctor to evaluate simultaneously medical data of numerous patients. Hence the doctor cannot correctly prioritize his efforts and consider different patients' needs within a group (51).

Success of treating NCD patients also depends on the ability of the treatment program to change patient's daily behavior affecting his/her health. This can apply to eating habits, physical exercise, smoking, etc. Patient's compliance is much influenced by subjective comprehension of his condition, education level, beliefs, incentives, etc. Design of treatment or follow-up plan should consider all these factors to provide maximum efficiency (47).

Patient's compliance is also connected with psychological resistance which can be due to temporary relief of symptoms or with too high demand that the treatment program imposes on habitual life style (and therefore, too much efforts that are required to change behavior). Consequently, the program should involve adequate feedback from the patient, detect resistance and correct the program to increase compliance (47).

In cases where the diagnosis is not quite clear, dynamic follow-up is necessary to better understand the patient's condition and to timely correct individual treatment plan.

The existing national and international clinical guidelines on NCD prevention provide very little individualization; they cannot motivate patients to change their life style—for example, by giving forecast of expected lifespan and medical costs (52).

Several patent applications underline the need in the instruments that would allow the patient to measure, register and analyze independently vital health indicators and risk factors, and also use them to monitor the efficacy of administered interventions (53).

Optimum health management in general is a complex problem that requires integration of data from multiple diverse sources. In particular, in addition to many types of health parameters, to continuously updated knowledge about human physiology and pathology, optimal management requires information about the best medical practices in various fields of medicine. The current health care system cannot provide patient-centered health management over the whole lifespan. Today's healthcare providers operate “on demand,” and therefore can offer only a small part of the whole continuum of care that an individual may need. Today there is no system or single provider of integral, holistic health management encompassing the whole care continuum (68). Preventive measures to reduce NCD risks are recognized clinically and cost effective; however, their wide introduction is difficult due to problems in patients' comprehension of professional clinical guidelines and lack of individualization (47).

According to a 2012 study by the Bipartisan Policy Center, healthy behaviors are the main determinants of the health state of the average US citizen (50%), with environmental and genetics contributing another 20% each, and access to health care determines only 10%. However, medical services dominate the health-related spending of American people (88%), with spending aimed at promoting healthy behaviors only totaling 4% on average (68).

### Evolution of Particular Technical Solutions in Health Management Systems: Disease-Centered Reactive Model vs. Person/Patient-Centered Preventive Model of Care

The evolution of proposed solutions and practices in automated health management systems over the last two decades can be attributed to the following factors: (1) development of digital and telecommunication technologies as tools necessary for scaling any medical technology; (2) better understanding of lifestyle-related factors as determinants of disease progression and health improvement, and emerging evidence that supports this idea; (3) global adoption of patient/person-centered instruments, interventions and models of care as a promising way to increase cost effectiveness. That is, in terms of effectiveness patient-centered health management systems have a significantly higher potential to improve the existing healthcare system, as can be seen from Table 3.

**Table 3:** Comparison of key parameters of diagnosis-centered and person-centered healthcare models relevant for design of AHMS.

**Parameter**	**The “old, reactive,” diagnosis-centered model**	**The “new, proactive,” patient-centered model**
The part of health continuum where major measures of health management take place	Acute disease or chronic disease with significant clinical representation (=a prerequisite for diagnosis)	Same as in the “old” model + patients with early premorbid health conditions and generally healthy people (with risk factors)
How the strategy of health management considers the root causes of NCD onset and progression	Almost fail to consider in everyday practice	Health is determined by factors that influence a person daily over a long term
Correlation with continuum of care	Fragmented care: 1. Emergency care 2. Hi-tech hospital care for complications of an NCD 3. Medical rehabilitation (short-term, hi-tech) 4. Outpatient follow-up for certain patient categories	Integrated care that is involved in addition to the fragments of the “old” model (pp. 1–4): - early premorbid prevention; - long-term rehabilitation in home setting; - long-term follow-up in lifestyle correction
The nature of interaction between patient and health professional	Usually one-time or occasional	Continuous and planned (regular)
Agents of health management	1. Patient 2. Doctor	1. Patient 2. Doctor 3. “Healthcare provider” who organizes interaction
Who initiates interaction and why	1. Patient, due to pain or discomfort 2. Planned check-ups (professional or annual)	Patient, due to pain or discomfort; planned reminders from “provider” Planned mandatory check-ups
Who makes decisions about goals and plan of health management	The doctor; the patient signs an “informed consent”	The patient him/herself with assistance and information support from the doctor
How decisions, goals, and action plan relate to medical diagnosis	Standards of examination and care are determined by medical diagnosis	Maximum personalization; adapted to individual physiology, psychology, social context, and lifestyle
Doctor's role	The key: decision making, drawing the plan of examination and treatment, legal liability	Expert, consultant, and coach in health management
The role of user (patient)	Passive: turn to the doctor in time; follow recommendations; pay for services; actual lack of responsibility for one's unhealthy behavior	The Key: goal setting, decision making, implementation of health management plan through behavior change; responsibility
Major instruments of health management	Medications; surgical procedures and operations	“Planning and control of following the therapeutic regimen and/or modifiable lifestyle factors”: 1. Healthy food 2. Sufficient motion and exercise 3. Reducing toxin exposure 4. High quality sleep and rest 5. Effective stress management 6. Healthy relationships
Major sources of information about patient's health condition	Doctor (results of examination), laboratory and instrumental examinations	Patient: questionnaires, case monitoring with wearable devices; the “old” sources if necessary
Place where care is provided	Separate medical organizations not interacting with each other	An integrated information space (ecosystem), contributed by a “family” doctor and “provider” (supports the infrastructure)
Place where health information (health record) is stored	Medical organizations; generally isolated from the patient; access is usually closed for patient and other health professionals	The patient him/herself (through User Account); patient opens access to health professionals
Major mode of patient-doctor interaction	Physical contact in doctor's office	Continuous (planned) and remote, including telemedical instruments; personal contact if necessary
Automation instruments	“Traditional” MIS comprising CRM (customer relation management) and ERP (enterprise resource planning): 1. Electronic medical record; records of medical services 2. Auxiliary services (pharmacy, laboratory) 3. Accounting and finances	1. User accounts of patient (consumer), doctor (service supplier), and “provider” (responsible for infrastructure) 2. Accounts are integrated with “traditional” MIS and telemedical instruments

Patent literature reflects well this evolution; however, a major factor whether a particular technical solution would be put into practice or not, lies beyond technology: we have found that two most adequate solutions have not been commercialized.

Analysis of relevance of 36 patents and patent applications to criteria of self-contained ASHM is summarized in Table 1; each of the relevant inventions is further discussed in detail with comments.

The first relevant patent that we have found [(47), application filed in 2000] describes an interactive computerized method and system for determining the risk of developing a disease, and consequences of the disease with the use of questionnaires; it also enables monitoring of personal health state. The method involves identification and modification of risk factors, including nutrition, through patient education. The method is based on the use of official clinical guidelines, and therefore is able to identify only a small portion of lifestyle-related risks; it cannot be applied to many common functional problems, especially before an NCD diagnosis can be confirmed. The method is aimed at identifying the risks of particular diseases, whereas one lifestyle factor can increase the risk of multiple diseases, and one disease can be determined by multiple factors. From practical standpoint, the proposed method only partially covers certain cardiovascular diseases (coronary heart disease—CHD). It implies mandatory interaction with a doctor, since it contains no particular recommendations on lifestyle modification; that is, it cannot serve as a decision support system for an individual in health management. The proposed method doesn't enable early detection, monitoring and correction of not only common functional conditions, but also for CHD itself. In particular, it fails to provide a complete list of questions for questionnaire, and an algorithm of their analysis. Essentially, the proposed method is an algorithm of diagnosing CHD as an established condition; it fails to consider contemporary knowledge of the whole range of factors determining development and outcome of even CHD.

The next patent by Japanese authors [(52), priority of 2001, patent owner Hitachi, Japan] describes a system of health screening and data processing that provides an estimate of expected life span, future health care expenses, along with recommendations (a health management plan) for behavior modifications intended to extend the life span and reduce future expenses. In screening the system considers excessive weight, high blood pressure, dyslipidemia, high blood glucose, and uric acid. Out of all lifestyle factors the system considers smoking, alcohol consumption and physical exercise. Health management plan includes low calorie diet, quitting smoking, exercise (without any details), and repeated screening. The system is designed to inform the user how the proposed plan would extend the patient's life and reduce health care expenses; it is expected that this information should motivate the individual for behavior modification. The proposed system can be used in medical insurance, but in terms of patient-centered health management it only considers some of important lifestyle factors and relies on laboratory tests which are marginally sensitive to premorbid health conditions.

Patent authored by Pascucci and Pascucci (53) provides a health management kit that includes portable devices for evaluation of health risks (measuring blood pressure, blood glucose and cholesterol) combined with a PC to interpret test results and provide recommendations. Obviously, this solution cannot enable adequate health management.

Patent by Rao et al. (66) describes an automated system for distant collection of information about health state, functional problems (signs and symptoms), and lifestyle factors including diet. It is designed to help in occasional food choices that would consider individual health. However, the system doesn't contain any algorithms for analyzing causality between functional problems and lifestyle factors; it doesn't provide any individual advice. Therefore, it cannot provide a method of early detection, monitoring and correction of functional disturbances, nor it enables health management.

A similar application was filed in Korea (30): an Internet-based U-health care system using biometric and patient-reported data to enable remote specialist decision making. It provides only principles and very general description of the system, which makes it impossible to implement it directly. Its most probable primary utility is early detection and prevention of emergency health conditions.

In 2010, two patents were granted for methods of automated remote monitoring of health state and health management in patients with medical diagnosis (chronic disease like diabetes and coronary heart disease, CHD) with the use of stationary and wearable devices (46, 48). They are currently owned by Health Hero Network (USA, https://www.gohealthhero.com/). These methods can detect abnormality of physiological parameters and give recommendations on medication and correction of lifestyle factors that influence those parameters; recommendations are based on official clinical guidelines. The described systems improve patients' awareness of their conditions and of the ways to prevent disease complications, and also improve compliance. These methods have the following shortcomings: they cannot be applied until a medical diagnosis is established (i.e., they are useless in most people with early functional impairments); official guidelines fail to consider individual causal relations between lifestyle factors and functional impairments; they imply purchase of a device (which make them less affordable); they require a doctor.

Another patent of Health Hero Network (54) covers a virtual trainer system that can be considered a part of automated health management systems responsible for physical exercise. The trainer allows to improve skills in physical exercises by simulating a professional coach. The next patent of the same company (55) focuses on motivating the user to engage in physical exercise with a telemonitoring system and a digital assistant. The both systems can cover only a small part of functionality necessary in a complete health management system.

Another service was claimed as a health management system (67): it allows the patient to upload description of his/her complex pharmacotherapy on the web site profile and open access to it for different doctors for expertise and corrections. Obviously, this solution cannot be a complete health management system, since it solves a very narrow technical task for a small patient category exposed to polypharmacy and running a higher risk of undesirable drug interactions and adverse effects. In fact, this is rather a disease management than a health management system.

A patent by Rao and Rao (39) proposed an organizational solution for managing health of multiple users, especially in rural regions, with poor access to health care. The invention provides simple access to medical information, opinions of doctors and experts, without additional infrastructure costs. It involves the use of questionnaires and medical devices to remotely collect and transmit health data in automated mode (through mobile devices), a coaching module, and presentation of information content related to lifestyle. In essence, the patent describes telemedicine-based health management with the use of remote server accessed from mobile devices. This patent covers only organizational solution, and lacks substantial aspects necessary to design a complete health management system, including the method of screening, early detection and correction. It doesn't enable a method of health management.

In 2012–2014, Abbott Diabetes Care patented several technical solutions for automated collection and processing of blood glucose data from diabetic patients; one of them is presented as “a system for managing treatment of a particular health condition via a phone device” (56). The system involves a glucose measuring unit connected to a mobile PC or phone (including cordless connection); it enables automatic transfer of the readings to data base for storage and processing. The device can remind the patient to input data (including data on taken medications), can transfer both objective readings and subjective indices [for example, symptoms of stress and depression—(57)], and even information on daily activities (including nutrition, exercises, etc.) along with time mark when the data are read. The system can show correlation between symptoms, behavior indicators, intake of medications, and laboratory readings (58). Another technical solution (59) allows creating and storing in the mobile phone daily individual plan of daily regimen with reminders about necessary activities. Based on the all mentioned solutions, the authors present a principle of managing the treatment of a certain disease (60). The eventual system can integrate the functions of measuring, processing and transferring disease indicators and certain lifestyle indicators; it allows monitoring of signs and symptoms bound to treatment regimen and lifestyle. The proposed system can provide more effective monitoring of particular signs or symptoms and be useful to analyze dynamics and efficacy of treatment for an existing chronic disease. However, all mentioned patents provide only organization aspects and lack essential medical details. Such system doesn't allow health management in early premorbid conditions and fails to consider the whole range of factors relevant for health management.

Later, in 2015, an affiliated company, Abbott Laboratories, patented a system for managing healthcare of patients with chronic diseases (61). Their method involves creating and maintaining a register of patients (with patient profile, an analog of electronic medical record), where each patient undergo screening and planned regular examinations; then based on dynamics of the key disease progression indices, a plan of treatment or follow-up is proposed in agreement with professional guidelines. Patient profile can be updated, with corresponding update of individual treatment plan. The system is designed to coordinate medical care that the patient receives from different providers, to consolidate in one database all patient's medical records, and reduce overall healthcare costs. It doesn't suit personalized health management of patients with early premorbid health conditions (before chronic disease diagnosis is confirmed); as described, the system fail to consider the role of lifestyle factors in onset and progression of chronic disease; the patent covers only a system applied to diabetes.

Philips company (the Netherlands) received a patent on health management system designed for patients with chronic disease after discharge from the hospital. It allows remote long-term follow-up and support in home setting with the use of video content prepared beforehand to inform the patient about on-site rehabilitation program (51). The system allows individualized presentation of the content depending on specific rehabilitation goals for the particular patient. A separate content module with a set of sessions is prepared for a specific possible health management goal. The modules are offered to the patient depending on the current goals; the sessions are presented in interactive mode, with feedback from the patient. Each next session is selected depending on the feedback. The system in general provides an effective individualized information support for patients with chronic disease transitioning from hospital-provided care to self-care. However, it fails to describe planned continuous evaluation of patient's signs and symptoms, and lifestyle factors that determine the course of chronic disease. The more so, it doesn't fit for early premorbid health conditions. That is, the proposed technical solution can only be useful as a local element of a more complete health management system.

Keas (USA, https://www.welltok.com/), a consumer activation company and one of the leaders in promoting healthy lifestyle, patented in 2014 a method and a health management system with gamification elements (62). The system involves a community (social network) of people mastering specific healthy behaviors; a type of competition is set between community members, with assigning achievement score for completed tasks or mastered skills within a recommended plan. The described technical solution introduces gamification principles in transition to healthy lifestyle, but it fails to propose specific content for either evaluation of the user's needs (like screening, monitoring) or for proposing specific plans of behavior modification (correction of existing problems or risk factors). Therefore, the patent doesn't enable a complete health management system.

An application filed by Siemens Healthcare GmbH (68) proposes a sophisticated method and system for automated “holistic management of health.” Medical data are fed through sensors, visualization instruments, laboratory tests, or examination; they form a base for individual computational physiology model; then a holistic health management plan for the individual is generated based on the current health state of the individual using a trained intelligent artificial agent. The key feature is a computational physiology model (a complex of models for particular body systems and processes) which is corrected and simulated based on actual patient's data, and is used to train the artificial intelligence (AI). By “holistic health management plan” the authors mean consultations with health professionals, non-prescription medications, individual nutrition advice, plan of exercise (standard or adapted individually), and also laboratory tests and prescription drugs as necessary. The application involves AI based on machine learning algorithms, deep learning architecture or deep neural network. A distinctive feature of the application is focus on a digital model of physiological processes and machine learning-based AI. However, these instruments *per se* cannot allow transition from reactive diagnosis-centered model to preventive person/patient-centered model of health management, if they don't focus on lifestyle factors and patient's key role in behavior modification. Therefore, this application's potential to solve the problems of health management can be estimated as very modest. Also, it fails to disclose many substantial features necessary to develop a complete health management system.

Taken together, all the above solutions are more in line with the model of reactive disease management than proactive health management; they suit for only individuals with established NCD diagnosis and generally follow formal clinical guidelines, without actual personalization. Therefore, their potential to solve the real problems of health management is very modest.

The first patent focused on a personalized lifestyle-related approach (20) was filed in 1997 and later turned into a very successful business project, www.sharecare.com (online test for ‘real age’ and related individual wellness programs). Although the patent doesn't directly claim a health management system, it describes its key elements: measuring of wellness, examination of its personal determinants, selection of the relevant lifestyle modifications, and motivation for behavior change.

An application by Japanese authors filed in 2004 (25) describes an automated HMS for lifestyle-related diseases and focused on evaluating lifestyle factors and giving the patient an opportunity to select from a plurality of possible changes in daily activities—that is, on involving the patient in managing his/her own health and improving the quality of life. The document lacks specific details of data acquisition and decision making algorithms which prevents its practical use.

Another attempt to base a health management system on daily lifestyle factors was made in application filed in 2005 by Bergantino (34). It describes a method of health management through analysis of individual's nutrition and development of an optimal nutrition plan. This method considers only one lifestyle factor, nutrition; it doesn't allow detection of functional problems, analysis of cause-effect relations between individual lifestyle factors (including nutrition) and functional problems; it doesn't support decision making in correction through lifestyle factors. Therefore, this application couldn't become a health management system.

The next application by Gerjets et al. (37) was filed in 2005 by Center U LLC. This is a physician directed method (and system) of computerized comprehensive health assessment and development of a science-based individual health improvement plan. In collecting information it uses a dynamically changing patient questionnaire reflecting functional scores and lifestyle factors, patient history, and laboratory tests, including genetic factors. Based on the questionnaire, the expert system draws conclusion on patient's functional impairments and develops an individual plan of nutrition and lifestyle. The expert system uses “PubMed” electronic database of the National Library of Medicine. The system is only useful to support decision making by physician. The authors didn't design it for automated screening or monitoring, evaluation of risks of the most common functional impairments, or support decision making by the patient in managing his/her personal health. Most importantly, the application fails to disclose specific algorithms of the expert system, which makes it useless in practice. Eight years later the same company made another attempt to patent a health management system (see below).

In 2005 medical diagnostic company Microlife (https://www.microlife.com/, Switzerland) filed an application covering a method and system for teaching and guiding an individual in changing the lifestyle (17). Its author was company founder, a citizen of Taiwan. The application is currently abandoned. The system's key elements are (1) collection of individual information about health, (2) informing the patient about causal relations between his/her signs and symptoms (markers of disease) and individual behaviors, and (3) teaching principles and algorithms of healthy lifestyle. The method involves remote monitoring of health, a school for healthier life (teaching healthy lifestyle), and holistic methods of treatment and health improvement. The application gives a broad overview of the system, with its basic principles and some particular details of diagnosing diseases. However, it lacks certain important technical details necessary to use this approach in modifying the lifestyle factors. In particular, information about functional impairments and lifestyle factors that should be fed to the system is given in a very general way; no clues are provided as to how to analyze this information and develop individual recommendations. Even with the mentioned shortcomings, this system can be considered as the most complete and adequate health management system out of all that were described in applications filed to the USPTO. Despite relative simplicity, this system has not been put into practice.

A patent filed in 2006 (26) describes a method of choosing lifestyle recommendations based on person's lifestyle and healthcare information obtained from a special aggregator. This is a sound biopsychosocial approach to health involving financial aspects, although the patent lacks details of data analysis, decision making algorithms and practical results, as well as consideration of some important factors like sleep and stress management.

More active patenting of systems focused on personalized and preventive health care through lifestyle modification started since about 2010.

In 2010 Pathway Genomics Corporation (https://www.pathway.com/) filed an application covering a health management system (apparatus) designed primarily to manage excessive weight (33). It is based on evaluation of individual inherited risks related to genetic markers, and on providing personalized plans of diet, nutrition and exercise. Today this system is marketed as a line of products that involves evaluation of dozens of genetic polymorphisms. In Russia a similar system is introduced by MyGenetics company (https://mygenetics.ru/) as a “genetic passport” with evaluation of 33 gene polymorphisms associated with chronic diseases. Essentially this system provides not health management but evaluation of risks of chronic diseases based on polymorphisms of genes implicated in NCDs. However, NCDs have multifactorial nature, with leading role of daily lifestyle factors, and genetics as a predisposing factor. Individual lifestyle factors interact to influence activity of multiple individual genes. Therefore, the proposed technical solution cannot be a self-contained health management system, but only supplement holistic patient-centered health management.

A patent was granted in 2017 to an application of 2010 that covers a method and computer program for remote automated personalized management of weight, lifestyle and/or disease (32). It claims integrated personalized “disease management and behavior modification” with the use of coaching. This method involves collection of information about functional impairments and patient's lifestyle with the use of questionnaires; it also evaluates person's readiness for behavior change and presence of health management skills. The system involves a platform for coaching: instruments to select a coach and communicate with him. Coaching is a method of consulting and training where in reaching a specific goal the coach instead of giving advice or firm recommendations is looking for a solution in cooperation with the client. The described method has a mandatory step of testing 3 genes related to obesity risk. The algorithm of health management according to the invention includes: (1) patient categorization based on information about personality attributes, nutrition, physical activity, and genetic profile; (2) selection (based on categorization) of one out of several unified programs to modify behavior (primarily, nutrition and physical exercise); (3) selection of a coach. Unified programs can be updated based on new scientific findings. The method allows storage of information about the patient and his progress in the selected program, and analysis of programs' and coaches' efficiency. Shortcomings of the method: it actually doesn't allow identification and evaluation of functional impairments, presence of risks of chronic diseases other than obesity. It fails to assess the whole range of all important lifestyle factors; it fails to provide automated design of recommendation on lifestyle correction; its functioning is dependent on personal coaches. As the result, the system doesn't provide automated health management and support of patient's decisions on managing personal health.

In 2015 Total Wellness Clinic (USA) filed an application covering a health management method based on the principles of integrative medicine [(21), application is abandoned]. It is based on an understanding that a person's functional state can be improved not only in an established disease but in any suboptimal health condition. This is possible with implementation of an integrative approach considering person's biochemical individuality and modified lifestyle factors. The application describes a method of health management (including conditions without an established disease) that combines Western medicine, Chinese medicine, Functional medicine, and bio-energy medicine. Based on initial evaluation of health, patient is offered treatment with various methods adapted to patient's individual circumstances. This method can only be practiced by a highly qualified professional. It doesn't fit for automated screening, monitoring and correction of functional disturbances in many patients.

In 2017, Better Therapeutics LLC (USA) filed patent application claiming a method and system for managing lifestyle and health interventions (31). It involves managing a lifestyle-related disease through remote interaction with a patient using digital interface. The method involves collection of information about patient's symptoms and lifestyle factors, and providing individual recommendations on lifestyle change to achieve a target condition for the patient's disease. This solution is implemented in a popular and effective online service promoting patient-centered concept of “lifestyle medicine” (https://www.bettertherapeutics.io/). It has certain limitations: it doesn't allow early detection of functional impairments since it covers only patients with already established diagnosis of a cardiometabolic disease. Description of the invention doesn't contain algorithm of revealing the causal relations between patient's symptoms and lifestyle factors. Therefore, it cannot enable an automated decision making support system for patient's health management.

In 2016, OutcomeMD Inc. claimed a method of automated determining a wellness score with the use of medical questionnaires (35). It enables evaluation of the user's current state (severity of functional impairments), changes over time, treatment effectiveness; it can also inform the patient about study results and give an interpretation. The application mentions numerous special medical questionnaires designed for hospital setting or under doctor's control. An online service based on the proposed method (https://www.outcomemd.com/) is claimed as an Outcome Management System designed for doctors of various specialties and using patient-reported outcomes. The method can considerably increase the effectiveness of health care. However, it doesn't imply the patient's leading role in managing his/her own health: it doesn't allow automated screening and early detection of functional impairments; the application doesn't describe analysis of the injected data. The method ignores analysis of lifestyle factors, therefore the user cannot identify the causes of functional impairments and cannot receive lifestyle-based recommendations how to correct the problems.

In 2013 Center U LLC made another attempt to patent a health management system in the format of a method for analysis (interpretation) of data received from the patient (36). The method involves filling health state and lifestyle questionnaires, automated calculation of functional scores (including their changes over time) and providing recommendations of lifestyle correction. Description contains very thoughtful ideas about insufficient use of lifestyle factors in the current doctors' practice and decision making. However, the application is focused in local formal algorithms of data analysis; it fails to propose a model of causal relations between the patient's symptoms and lifestyle factors that can be used in automated selection of recommendations on behavior change. There are no algorithms for practical implementation of the method. Therefore, the system either requires a doctor or cannot be implemented at all.

A patient-centered solution was proposed in two applications by Chinese authors. They involve the use of Traditional Chinese Medicine (TCM) to identify health problems, patient's constitution, and choose necessary preventive interventions (27, 28). TCM is an integrated self-sufficient system, however, the proposed solutions actually represent a tool for TCM doctor for data acquisition, decision making support, and patient follow-up; the set of data and algorithms of analysis and decision making are non-transparent. Another Chinese application (40) discloses a telemedicine system for automated data acquisition and involvement of patient's relatives and friends, without clear decision making algorithms and results.

Two patent documents by International Business Machines Corp disclose a personalized health care management system that pioneers with a focus on changing the user's behavior (18). The application discloses a method of processing the user's data on health and behavior patterns (lifestyle factors); the data can be obtained with any wearable sensors and behavior trackers, from electronic medical record, and also from questionnaires. User data also cover available resources and other details of life that determine the context for possible lifestyle correction; these data are obtained through analysis of patient's behavior in social networks, and also from locations associated from his/her daily lifestyle, information about environment quality of home and job, availability of local infrastructure relevant for health management, patient's membership in loyalty programs, the history of purchases and ordered services, etc. Through extensive analysis of information about patient's actual behavior patterns and lifestyle, it becomes possible to compare the actual and potential optimal patterns, identify deviations and propose advice in minimizing those deviations. For example, based on analysis of daily locations and available local sources of healthy food, places for physical exercises and recreation, the system could propose daily behavior patterns that would enable optimal lifestyle. Health management plan would actually represent a list of goals and specific behaviors to reach those goals. Personalization of the plan is due to maximum consideration of the user's lifestyle context and daily behavior patterns. The described system can enable very high personalization of health management. However, its significant shortcoming is complexity in receiving data from third-party sources and in integrating those data in a single system. For effective operation, this system would require an expensive hi-tech digital infrastructure; the system is very time- and resource-consuming. On the other hand, the system fails to duly consider certain important lifestyle factors which are less available for tracking: for example, the level of emotional stress and user's ability to manage stress. The application also ignores the aspects of lifestyle related to intrapersonal communications and spiritual dimensions of health. The second very close patent of the same assignee (19) implies that personalized patient care plan is based on both health record and any exogenous lifestyle-related data; the plan involves behavior modification to minimize the effects of exogenous conditions. The patent also involves machine learning and evaluation of patient's compliance. Limitations of this solution are similar to those of (18).

## Discussion

Above we have analyzed 17 non-patent sources and 36 patents and patent applications filed worldwide that claim and actually describe a personal health management system of different level of self-sufficiency and workability.

At least 10 of the mentioned applications have been implemented as commercial systems or services. Unfortunately, the two most sound (in our opinion) systems have not been put into practice, (17) and (18).

As a motivation to develop a health management system, most authors point at the problems and shortcomings of today's health care (health management) which are naturally inherent in the currently prevailing paradigm and model of medicine that can be termed as “reactive” and “diagnosis-centered.” Some authors propose local improvement and modifications within the currently prevailing model, while others promote a new person/patient-centered model (70), more suitable for overcoming the epidemic of chronic diseases.

### Conceptual Context

Below we would like to build a concept of an “ideal” workable ASHM based on analysis of the above-mentioned sources and on our understanding of what health management is and should be.

Management as a universal process deals with information in the first place, and therefore most authors take into account three major natural requirements for a health management system:

1) The system should encompass the complete cycle of processing information about patient's health and behavior (acquisition, analysis, storage, use for planning the interventions, and receiving the feedback on intervention results);
2) Operations with information should be automated as much as possible;
3) The system should be scalable.

In designing automated systems for personalized health management (ASHM), most authors proceed from the following three important prerequisites:

Prerequisite 1. The target audience that needs effective health management most of all is patients with NCDs and other chronic health conditions.

Prerequisite 2. Health management should consider continuity of care and represent a continuum of care. The latter term was introduced in the late 1980s (7) to describe the continuous range of all potential measures designed to meet all needs of an individual in recovery, support and improvement of health. Continuity includes three aspects (8):

(1) Informational continuity—The use of information on past events and personal circumstances to make current care appropriate for each individual
(2) Management continuity—A consistent and coherent approach to the management of a health condition that is responsive to a patient's changing needs
(3) Relational continuity—An ongoing therapeutic relationship between a patient and one or more providers

The overall goal of care continuum should be a tangible result for patient in the form of significant improvement of health.

Continuum of care can be described as the following series of measures [(8, 9), with modifications]:

At an early premorbid stage: (1) informing about the principles of healthy lifestyle and teaching algorithms of health management; (2) screening for early signs of health problems, detection and monitoring of premorbid conditions; (3) prevention of chronic diseases through correction of lifestyle factors.

At the stage of non-complicated chronic disease: (4) Examination in outpatient and hospital setting; (5) Planned interventions in outpatient and hospital settings; (6) Long-term follow-up at home to prevent disease complications, stop progression, and probably reverse the disease through correction of lifestyle.

At the stage of complicated chronic disease and disability: (7) Examination and interventions in out-patient and hospital settings, including states of emergency; (8) Rehabilitation by interdisciplinary team, in hospital and home settings; (9) Long-term follow-up at home, including the use of home care technologies.

At all stages of health and disease continuum: (10) informing about available lifestyle interventions and teaching health management algorithms.

Many inventors underline that today's healthcare model provides only a narrow range of services mentioned in the described continuum of care.

Prerequisite 3. Health management implies active patient's role and regular continuous interaction (follow-up) between patient and healthcare professional.

Ideally, care should be practiced as not only patient-centered but also person-centered care. This model implies collaboration between doctor and patient in development and implementation of personalized program; only collaboration can provide the patient with results he/she needs in the most cost-effective manner.

It is obvious that a health management system has to be automated with the use of a medical information system (MIS) on the one hand, and should be accessible from home or other place where the patient spends a major part of his/her time. A good review of problems and opportunities in the use of MIS in home setting can be found in a paper by Stolee et al. (71). Among other, this paper mentions the following barriers for automation with the use of Electronic Medical Records (EMR): the system is not focused on the client (the final consumer); the system doesn't collect information relevant for health management (“the right information”); the system is very labor consuming; one patient's data are scattered over MIS of different medical organizations.

The mentioned problems reflect the disadvantages of today's diagnosis-centered reactive model of healthcare: it features key role, burden and responsibility of the doctor (medical professional); it uses too little information about patient's lifestyle factors, and it uses too little patient's natural resources (time and attention). As the result, the healthcare systems struggles with shortage of resources (access to doctors), while the most potentially effective tools of health management (lifestyle interventions) appear the least used tools compared to pharmacotherapy and surgery.

These problems can be overcome through active introduction of patient-centered approaches; one of them could be a cloud-based MIS integrating data from different medical organization, where the patient him/herself would be involved in input of data relevant for decision making.

### Integration of Common Technical Solutions Found in Literature: Design of AHMS and Different Healthcare Models

Common solutions and problems that they try to address are summarized earlier in Table 2. Here we would attempt to build on them an integral picture.

Let's look how the proposed technical solutions correspond to patients' need in health management, with continuum of care, and the nature of management.

For this purpose we will have to abstract away from particular disease and health conditions to look at the general pattern how personal health is changing over time [(2), with modifications]:

(1) from complete health →
(2) to early (premorbid) functional impairments →
(3) to development of an NCD →
(4) to NCD complications →
(5) to death from NCD complications.

As a rule, today's “reactive,” diagnosis-centered medicine comes into health management at stages (3) or (4); it primarily focuses on controlling the signs of NCD, and treats NCD complications in line with clinical standards (guidelines)—that is, actually without considering individual context and lifestyle factors that drive progression of NCD in a particular patient.

Here comes the first key conclusion about requirements for an AHMS that can be useful in NCD: it should be designed not within the framework of today's “reactive” diagnosis-centered healthcare model, but in line with proactive, person/patient-centered model. These two models are compared in Table 3.

### Design of AHMS and Patient's Needs

Depending on the health state, the users of AHMS (patients and healthy people) can have a wide range of needs in health management; in turn, the needs determine the relevant personalized measures. Table 4 sums up the needs of different user categories [based on common sense and continuum of care, see (8, 9)] and the corresponding functions of AHMS.

**Table 4:** The needs of AHMS users (=results that they want) and possible measures that can be automated in AHMS to feed those needs.

**The need (the objective)**	**The system of measures in AHMS**
1. Recover from NCD complications, improve quality of life	Organize timely treatment of NCD complications Organize long-term rehabilitation and follow-up at home Organize supply of necessary medications, means of rehabilitation and health improvement Teach methods of self-care and self-help, and practices of healthy lifestyle
2. Avoid NCD complications (if NCD is present)	Detect and monitor signs and symptoms of NCD; identify lifestyle factors that promote NCD progression Help in selecting treatment to control NCD symptoms and complications (including analysis of treatment effectiveness) Assist in changing everyday behaviors to healthy lifestyle in order to prevent NCD complications
3. Avoid onset of NCD (if risk factors and early premorbid functional problems are present)	Detect and monitor early premorbid functional impairments; identify NCD risk factors and lifestyle factors that promote onset of NCD Inform about causality of NCD and ways to prevent NCD through correction of lifestyle Assist in transition to healthy lifestyle to prevent NCD
4. Improve health	Identify lifestyle factors that can affect health and cause NCDs Inform about causality of NCD in lifestyle and ways to improve health through correction of lifestyle Assist in transition to healthy lifestyle to improve health

Each of the mentioned needs corresponds to different level of motivation: in general, people with more severe health problems (“more pain”) are more motivated to change their behavior. Person-centered model of healthcare involves modification of behavior patterns and individual habits, and therefore in designing AHMS one should bear in mind the internal logic of behavior change. The cycle of individual changes and the instruments of managing this process are described in detail by Prochaska et al. (72), and Fogg (73). Important instruments in this process are informing and raising awareness and mindfulness. From the viewpoint of personal change, health management should look like cycles of communication and interaction closely connected with natural cycles of person's behavior change.

What functions should AHMS have to implement the mentioned measures? Which of them were not considered or disclosed in the patents and applications that we analyzed above?

To address these questions, let's reconstruct the most plausible “trajectory” of AHMS user, the possible sequence of user's interactions with the system. This sequence and expected results are summarized in Table 5.

**Table 5:** Possible sequence of user's interactions with AHMS.

**Event**	**Expected result(s)**
1. The user registers in the system	Opportunities of the system are explained and instructions of its use are given User agreement is made; the user gives permission to store and process his/her data, and to send information, notifications and reminders
2. Identification of user's problems, needs, and risk factors	The user is informed and has a better awareness of his/her problems and needs Primary sorting is made in terms of user's need in a more thorough examination and/or intervention
3. Identification of potential sources of health problems in the user's lifestyle factors	The user is informed and has a better awareness of the most probable causes of health problems that he/she has Necessary information is collected to form a personalized health management program
4. A personal health management program is generated	Goals are set; available actions are chosen, with methods to implement them, timelines, necessary prerequisites (including resources) and participants (including health professionals)
5. Persona health management program is implemented	Exact steps are planned, and their implementation is controlled; necessary measures are taken to maintain motivation; current results are assessed
6. Evaluation of program effectiveness and goal setting for the next management cycle	Necessary feedback is received to adjust and fine tune health management strategy and tactics
7. Planned repeated evaluation of the current problems and needs	Continuity and integrity of health management is provided

After outlining the basic cycle of user interaction with AHMS, one can fill the system with the most relevant instruments and interventions for health management. Many of them are already present in current practices reviewed above, some remain to be developed and introduced.

Coming back to the descriptions of today's practices—health management systems given in the analyzed sources, there are three important points to add. None of the inventors has addressed at least the following key tasks of health management:

1. Consideration of all known significant lifestyle factors that may affect onset and progression of NCD in a particular patient (for example, combination of nutrition, chronic stress, lack of necessary physical exercise, lack of sleep, toxic burden, etc.).
2. Offering to the patient an algorithm of specific individualized recommendations how to change his/her lifestyle (for example, specific nutrition pattern, physical exercise pattern, stress management pattern, etc.).
3. Offering the patient an infrastructure supporting the proposed long-term monitoring and behavioral modifications (for example, in the form of tracking and/or coaching).

A lot of issues remain to be discussed about design of an optimum AHMS in line with person-centered healthcare model. This will be done in a separate paper. The authors feel that there are all prerequisites for creating an optimum AHMS already present in professional community and literature; it's time to bring them together like Lego bricks.

## Conclusions and Recommendations

Automated systems for personalized health management (AHMS) is a promising way to improve clinical and cost effectiveness of prevention and long-term follow-up of patients with NCDs.

Today's healthcare model focused on control of NCD symptoms can be defined as “reactive” and “diagnosis-centered”; its effectiveness is limited to acute diseases and emergencies, while AHMS is mostly required for prevention and long-term management of chronic health conditions.

Most technical solutions currently practiced or described in scientific and patent literature as AHMS only solve local tasks in health management; all diagnosis-centered AHMS have serious limitations and cannot provide effective and integrated solutions to most problems related to the global NCD epidemic.

To enable the best patient outcomes for chronic health conditions, an AHMS should be designed within the context of health continuum and continuum of care; it should consider the key role of lifestyle factors in the onset and progression of NCDs.

To be cost effective, an AHMS should be patient- or person-centered; it should imply patient's active role in managing one's own health: at least at the steps of collecting information, goal setting and implementation of lifestyle interventions.

To enable effective management of chronic health conditions, AHMS should consider all aspects of human health (physical, mental, and social), patient's needs and motivation; it should involve examination and modification of everyday behaviors.

Key prerequisites for developing and up-scaling an effective and ubiquitous (generally available) AHMS are: digital and telecommunication technologies based on Internet, mobile communication, portable and wearable devices and sensors; digital technologies should make major functions of AHMS available 24/7/365 and anywhere.

Automated algorithms of an effective AHMS should cover the following tasks: retrieving patient-centered data (questionnaires, wearable devices, etc.); evaluating self-reported symptoms, early predictors, risk factors and the need in detailed examination; providing decision support and individual advice on behavior modification; tracking the changes in behavior and symptoms of dysfunction.

Special focus in ASHM should be put on raising patient's awareness of cause-effect relations between everyday behavior and health problems, on informing him/her about available algorithms of lifestyle modification, and on patient's decision support system.

## Author Contributions

AM-P was responsible for the concept, data collection, analysis and interpretation, and drafting the article. DY contributed to the concept, data interpretation, critical revision of the article, and final approval of the version to be published. All authors contributed to the article and approved the submitted version.

## Conflict of Interest

The authors declare that the research was conducted in the absence of any commercial or financial relationships that could be construed as a potential conflict of interest.
